# Pilot *in vivo* studies on transcutaneous boiling histotripsy in porcine liver and kidney

**DOI:** 10.1038/s41598-019-56658-7

**Published:** 2019-12-27

**Authors:** Tatiana D. Khokhlova, George R. Schade, Yak-Nam Wang, Sergey V. Buravkov, Valeriy P. Chernikov, Julianna C. Simon, Frank Starr, Adam D. Maxwell, Michael R. Bailey, Wayne Kreider, Vera A. Khokhlova

**Affiliations:** 1 Division of Gastroenterology, Department of Medicine, University of Washington, Seattle, WA USA; 2 Center for Industrial and Medical Ultrasound, Applied Physics Laboratory, University of Washington, Seattle, WA USA; 3 Department of Urology, University of Washington School of Medicine, Seattle, WA USA; 4 Faculty of Fundamental Medicine, M.V. Lomonosov Moscow State University, Moscow, Russia; 5 Research Institute of Human Morphology, Moscow, Russia; 6 Physics Faculty, M.V. Lomonosov Moscow State University, Moscow, Russia

**Keywords:** Translational research, Acoustics

## Abstract

Boiling histotripsy (BH) is a High Intensity Focused Ultrasound (HIFU) method for precise mechanical disintegration of target tissue using millisecond-long pulses containing shocks. BH treatments with real-time ultrasound (US) guidance allowed by BH-generated bubbles were previously demonstrated *ex vivo* and *in vivo* in exposed porcine liver and small animals. Here, the feasibility of US-guided transabdominal and partially transcostal BH ablation of kidney and liver in an acute *in vivo* swine model was evaluated for 6 animals. BH parameters were: 1.5 MHz frequency, 5–30 pulses of 1–10 ms duration per focus, 1% duty cycle, peak acoustic powers 0.9–3.8 kW, sonication foci spaced 1–1.5 mm apart in a rectangular grid with 5–15 mm linear dimensions. In kidneys, well-demarcated volumetric BH lesions were generated without respiratory gating and renal medulla and collecting system were more resistant to BH than cortex. The treatment was accelerated 10-fold by using shorter BH pulses of larger peak power without affecting the quality of tissue fractionation. In liver, respiratory motion and aberrations from subcutaneous fat affected the treatment but increasing the peak power provided successful lesion generation. These data indicate BH is a promising technology for transabdominal and transcostal mechanical ablation of tumors in kidney and liver.

## Introduction

Renal cell carcinoma (RCC) and hepatocellular carcinoma (HCC) are two common solid organ tumors, combining for over 105,000 new U.S. cancer diagnoses in 2018^1^. With recent imaging advances leading to early diagnosis, an increasing number of patients now present with early-stage disease^2^. For patients with small localized tumors, minimally invasive focal therapies such as radiofrequency ablation^3,4^, cryotherapy^5,6^, and microwave ablation^7,8^ have been used as an alternative to surgical approaches.

While these technologies can be effective, recurrence rates tend to be higher than those for more traditional surgical approaches^9,10^. In addition, existing focal therapies are still invasive (percutaneous or laparoscopic) and have major limitations related to their reliance on thermally induced coagulative necrosis or freezing, which limits the size and location of treatments amenable to therapy. In larger tumors (>3–4 cm for RCC, >5 cm for HCC), uniform cell kill is generally not feasible and heat-sinking near vascular structures inhibits treatments near structures such as the renal sinus or large hepatic vessels. Moreover, heat diffusion can lead to unintended complications such as freezing/heating of the urinary collecting system^11^ or biliary system^4^, resulting in leaks and strictures.

High intensity focused ultrasound (HIFU) is an alternative, fully non-invasive energy modality for focal therapy. HIFU delivers a focused ultrasound beam within the body to locally affect the targeted site without damaging the intervening tissues^12^. HIFU is unique in its ability to produce tissue effects ranging from thermal to entirely non-thermal (i.e., mechanical), depending on the treatment regime employed^13^. Currently, the clinical HIFU sonication regimes implemented for most clinical applications, including HCC and RCC therapy, produce thermal ablation and are performed under MR thermometry or ultrasound imaging guidance^14–16^. However, due to their dependence on thermal effects, existing clinical HIFU systems have the same limitations as other existing thermal therapies.

Recent developments in HIFU technology have enabled several alternative approaches, collectively known as histotripsy, that mechanically fractionate or liquefy targeted tissue^17,18^. In these approaches, high amplitude ultrasound pulses are delivered at low duty cycle to produce and activate gas and vapor bubbles at the focus leading to disintegration of tissue into subcellular debris. Histotripsy treatments can be monitored in real time using conventional B-mode ultrasound, because the bubbles are reflective and appear as a hyperechoic region^19,20^. Additionally, progression of the treatment can be controlled as fractionation of tissue produces a hypoechoic cavity and the degree of hypoechogenicity corresponds to the degree of tissue destruction^21^. Another important characteristic of histotripsy is tissue selectivity: connective tissue structures (e.g. blood vessels, biliary structures, etc.) are more resistant to mechanical ablation than cells^22,23^. Further, the non-thermal nature of this approach removes limitations on treatment precision related to both heat sinking effects and collateral thermal damage associated with heat diffusion.

One histotripsy approach, termed boiling histotripsy (BH), uses milliseconds-long pulses of HIFU containing shock waves to heat tissue to boiling temperatures and produce a vapor bubble at the focus within each pulse^24^. The interaction between the rest of the pulse and the vapor cavity results in mechanical fractionation of tissue^25,26^. The feasibility and characterization of BH ablation outcomes have been previously demonstrated in *ex vivo* tissues and *in vivo* in exposed porcine liver and rat renal and subcutaneous tumors^20,27–29^. Here, we sought to evaluate the feasibility and safety of transcutaneous and transcostal volumetric BH ablation of porcine liver and kidney in acute pig studies. Further, we aimed to characterize the sensitivity of different renal and hepatic tissue structures (kidney cortex, medulla and collecting system, and hepatic lobules, ducts and vessels respectively) to different BH doses, defined here as the number of BH pulses applied per focus location.

## Results

**Figure 1:**
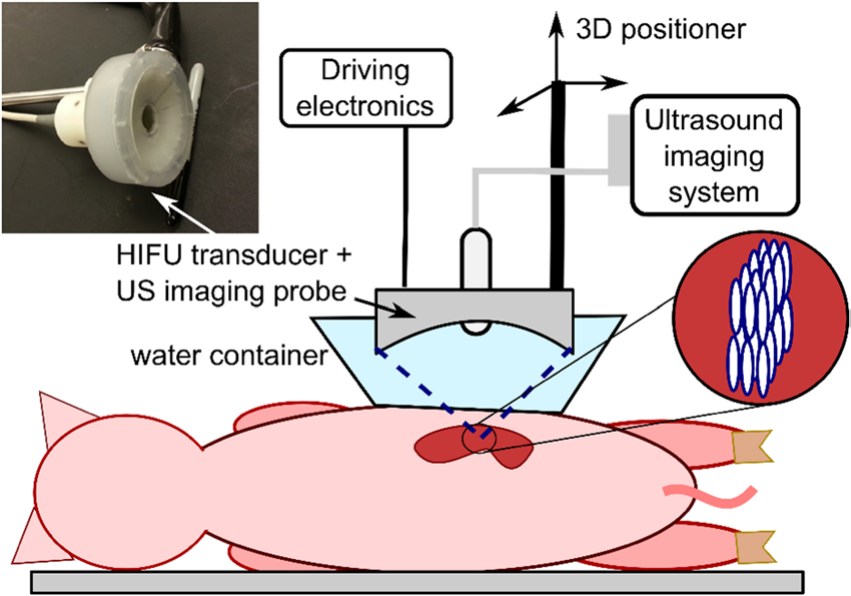
Illustration of the ultrasound image-guided boiling histotripsy (BH) approach. BH ablation treatments were performed using a custom-built 1.5 MHz HIFU transducer (12 sector elements, integrated with an ultrasound imaging probe in its central opening) for targeting and treatment guidance. Animals were sonicated in supine (liver) or lateral position (kidney, as shown). For acoustic coupling to the skin, the HIFU transducer was immersed in a water container with acoustically transparent window positioned over the animal. Volumetric BH lesions were produced by raster-scanning the transducer focus in three dimensions across the targeted area while delivering a specified treatment protocol at each focal spot (see inset).

To investigate the effects of volumetric BH ablation, we used N = 6 female domestic swine weighing 37–40 kg. Multiple volumetric lesions were produced in the bilateral kidneys (lateral position) and the liver (supine) using a 1.5 MHz custom-built HIFU transducer, as illustrated in Fig. 1. Targeting and treatment monitoring were performed with B-mode ultrasound imaging, with the imaging probe located in the central opening of the HIFU transducer. Higher resolution ultrasound imaging was performed immediately before and after BH treatment to evaluate treatment outcome in terms of lesion size and location. The animals were immediately euthanized after completing all treatments; the targeted tissues were examined grossly and processed for histology and transmission electron microscopy; the intervening and adjacent tissues were also examined grossly for damage.

### Feasibility and safety of kidney treatments

**Figure 2:**
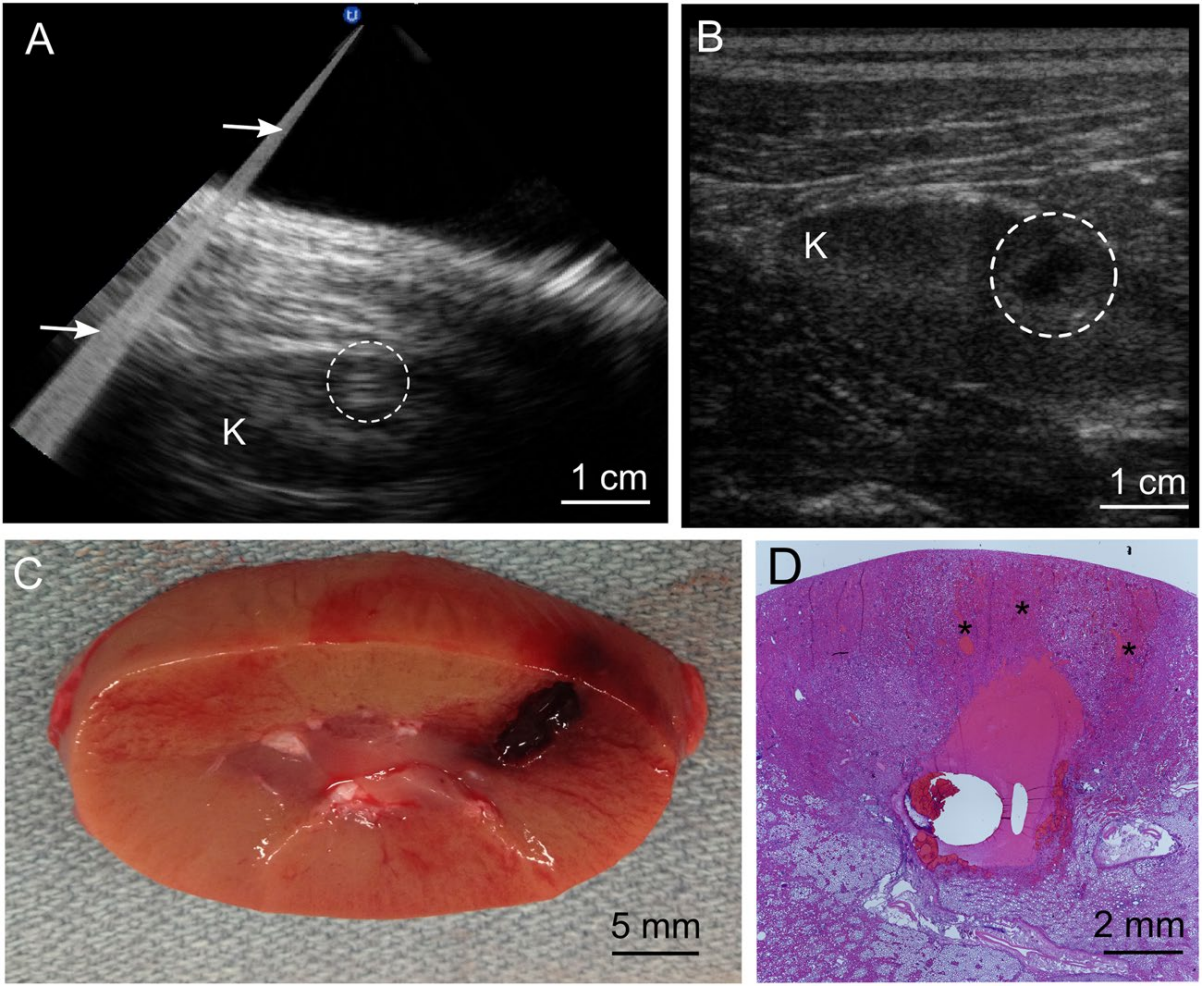
B-mode ultrasound imaging during and after BH treatment of the kidney provided high fidelity with gross view of the lesion. (**A**) Representative B-mode ultrasound image obtained during BH treatment of the kidney (K) showed a bright hyperechoic region (white dashed circle) at the focus which was used as feedback to indicate treatment initiation. Image artifacts from the backscattered BH pulse (arrows) did not prevent imaging of the target. (**B**) Post-treatment ultrasound evaluation of the treated area (white circle) revealed a clearly demarcated hypoechoic region corresponding in size and position to the liquefied region seen on gross inspection in. (**C**,**D**) Hematoxylin and eosin (H&E) stained histological section of the lesion obtained under the same exposure conditions containing sharply demarcated homogenized tissue. Regions of petechial hemorrhage (*)were seen prefocally, immediately adjacent to the homogenized ablation volume.

To assess if BH was feasible and to establish the output power required to achieve transcutaneous renal BH, single BH pulses were transmitted at increasing amplitudes until a hyperechoic region at the focus was observed, indicating the presence of vapor bubbles (Fig. 2A). This approach demonstrated that BH treatment to the lower pole was feasible in 11/12 kidneys (failure to produce vapor bubbles in the left kidney of one swine where the kidney was entirely retrocostal). Peak acoustic power outputs required to initiate entirely subcostal treatment at depths of 1.5–4.5 cm were 720–1600 W, which is 1.8–4 fold higher than that observed in preliminary experiments for exposed *ex vivo* tissue at the depth of 1 cm (400 W). Partially transcostal exposures, in which 30–40% of the beam obstructed by the ribs at depth of 3.1–3.7 cm required 1280–1920 W, i.e. 3.2–4.8 fold higher acoustic power compared to *ex vivo* exposures.

A total of N = 28 BH treatments in the 11 feasible kidneys were then conducted in which the renal cortex, medulla and sinus were targeted in 17, 12, and 10 of these treatments, respectively. Ttreatments in some exposures intentionally overlapped different regions of the kidney. To produce volumetric liquefied lesion, the HIFU focus was translated over the targeted region in a raster fashion using 1 mm steps in the transverse and cephalocaudal directions (up to a maximum of 15 mm) and 5 mm steps in the axial direction (up to two layers), delivering a set number of 10 ms BH pulses at each focus (5, 10, 15, 20 or 30 pulses). The size of an individual BH lesion at the same acoustic parameters is approximately 1–3 mm laterally and 6–10 mm axially, depending on the targeted tissue and the number of pulses delivered^30^. During all treatments, BH pulses produced bubbles that appeared as multiple moving hyperechoic regions on B-mode ultrasound image near the focus (Fig. 2A) with the gradual appearance of a hypoechoic cavity between pulses that persisted following completion of a treatment (Fig. 2B) and suggesting successful mechanical ablation. Respiratory motion did not appear to qualitatively affect targeting or imaging feedback in real time. At necropsy, gross inspection of the kidneys revealed voids in the cortex and medulla with regions of petechial hemorrhage located prefocally relatively to the homogenized volume of parenchyma (Fig. 2) corresponding to sites of treatment. Small clots were seen within the collecting system in 8/11 treated kidneys (Supplemental Fig. S1A,B). No gross evidence of collateral damage was observed within the beam path, however there was subtle bruising of the side wall immediately adjacent to the kidney following treatment of 1/11 kidneys in which a volume was created superficially within the cortex (Supplemental Fig. S1C).

### Histological evaluation of renal tissue following BH ablation

**Figure 3:**
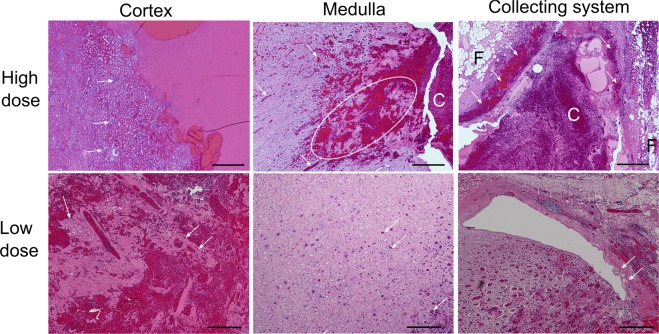
Different renal tissues - cortex, medulla and collecting system – showed differential sensitivities to BH ablation. Histological slides of the volumetric lesions stained with H&E were taken from the tissues subjected to the same BH dose, i.e. number of pulses delivered per focal spot. At the higher dose of 15–30 pulses per focal spot renal cortex is fully liquefied with no debris larger than 20 microns observed. White arrows indicate intact glomeruli adjacent to the liquefied region. In medulla, areas of disrupted, but not completely disintegrated connective tissue are observed (dashed circle), as well as blood within tubules (white arrows) that appears to flow towards collecting system and form a clot (C). Collecting system containing the clot has minimal observable damage in terms of bruising and sloughing of the urothelium (white arrows); the fat layer (F) appears intact. At low dose (5–10 pulses/spot) less tissue damage is noted within all three tissues. In the cortex, connective tissue fragments and structures with adjacent cells are observed within the lesion (white arrows). In medulla, only blood within the tubules is observed (white arrows), and very minor urothelium sloughing is seen in the collecting system (white arrows). The scale bar represents 500 microns.

On histologic assessment of lesions created in the renal cortex, all BH exposures at high doses (15–30 pulses/focal spot, 12/28 total exposures) produced completely homogenized tissue sharply demarcated from histologically normal untreated cortex (Fig. 3). Inspection of low dose cortical exposures (5–10 pulses/focal spot), revealed lesions containing fragments of intact stromal tissue structures (small caliber blood vessels and collagenous portions of renal tubules) interspersed with homogenized cellular debris, with sharp demarcation to completely normal-appearing cortex. Of note, in all cortical treatments, the overlying renal capsule, a thick fibrous layer investing the kidney– was completely intact, even in the 3 cases where the BH-liquefied area was immediately adjacent to the capsule (Supplementary Fig. S2A). In the medulla, small areas of homogenized tissue scattered through the targeted volume were noted at high dose exposures, while low dose exposures were apparent as only extravasated blood within the collecting ducts without apparent homogenized tissue. Additionally, lesion borders were not as well defined compared to the cortex. Inspection of central lesions, i.e. ones produced in the renal sinus and collecting system, demonstrated focal petechial hemorrhage within the wall of the collecting system and adjacent urothelial sloughing at the highest dose exposures (30 pulses) without disruption of the collagenous wall. At the low dose exposures, minor intermittent sloughing of the urothelium was noted with normal appearing adjacent collecting system wall. In all treatments targeting the center of the collecting system, liquefied renal sinus fat was visualized adjacent to the collecting system.

Based on the observations described above, respiratory variation did not appear to qualitatively impact the ability of BH to homogenize renal tissue or the precision of ablation. To further assess these effects, two separate lesions with identical volumes were planned to be generated within the cortex, such that the closest focal points in the respective 2D grids would be spaced 3 mm apart. Histologic inspection of the exposure outcome revealed two distinct volumetric lesions of homogenized cortex of approximately 0.3 cc volume each, separated by a thin ~1 mm bridge of normal appearing renal cortex (Supplementary Fig. S2B).

### Transmission electron microscopy of homogenized renal cortex

**Figure 4:**
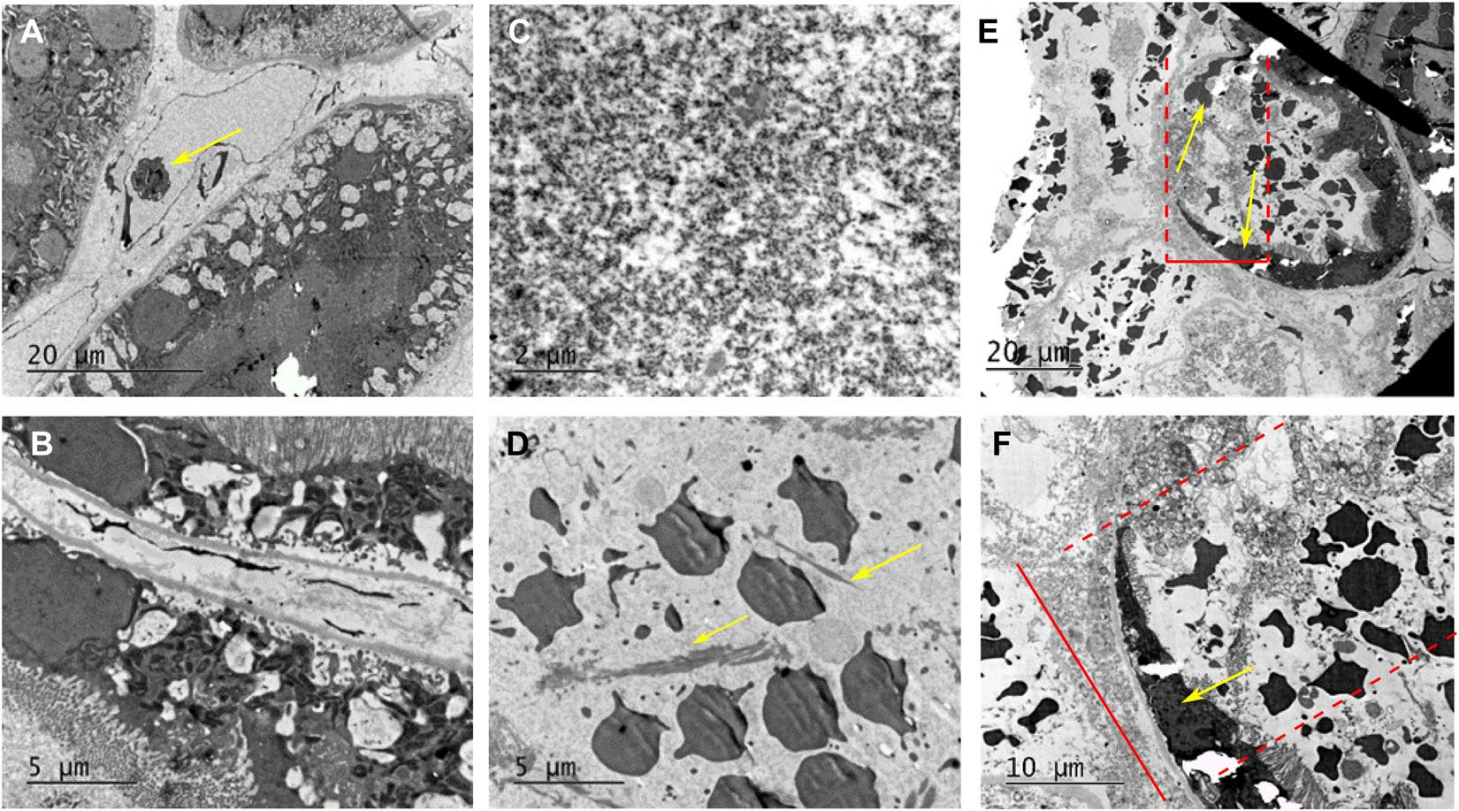
Transmission electron microscopy (TEM) of renal cortex ablated with BH. (**A**,**B**) TEM images of intact renal tissue adjacent to the BH lesion demonstrating proximal renal tubules and small caliber blood vessel with lymphocyte (arrow). (**C**,**D**) BH lesions containing a slurry of homogenized cellular debris <0.1 μm in size (**C**), occasional loose collagen fibers (arrows), and echinocytes (**D**). (**E**,**F**) At the lesion border, the transition zone width was 20 microns from fully intact to fully destroyed tissue (top red bar/dashed lines). Within the zone, the cells (arrows) are significantly damaged, without vili but containing some organelles (bottom).

To assess BH lesion contents and the lesion border at the ultrastructural level, areas of N = 4 liquefied lesions and adjacent tissues in renal cortex were collected and prepared for transmission electron microscopy (TEM). Representative TEM images of intact renal tissue, lesion contents and lesion border are shown in Fig. 4. Within intact renal cortex, proximal renal tubules and small caliber blood vessels were clearly visible as expected. Within lesions, regions of complete loss of structure with replacement of cells and organelles by electron dense sub-micron cellular debris with pockets of organelle ghosts released from otherwise completely destroyed cells. Additionally, fragmented collagen fibrils measuring up to 5–10 microns in length were observed intermixed with the cellular debris. Finally, intact erythrocytes were observed within the slurry of cellular debris consistent with post-treatment petechial hemorrhage. The border region between treated and untreated kidney measured 10–20 microns with clear transition from normal to completely disrupted cells over a region covering one to two tubule cells in thickness. In this border, intact tubule cells with sloughed villi giving way to cells with partially disrupted cell membranes but intact intracellular organelles were observed with normal and completely destroyed cells on either side. In some instances, a single cell was identified with a portion of it looking intact and a portion appearing nearly completely or completely destroyed. (Supplementary Fig. S3).

### Treatment acceleration strategy in renal cortex

Previous experiments^30^ have indicated that the lesion generated in *ex vivo* tissues by administering BH to a single focus is determined by the number of BH pulses delivered to that focus rather than total “HIFU on” time. In other words,a lesion created using 10 ms BH pulses will have the same size as that produced by using the same number of shorter BH pulses of larger amplitude, such that the pulse duration is longer than the time to initiate boiling. Accordingly, the use of shorter pulses delivered at higher pulse repetition frequency (PRF) (to maintain constant duty cycle) would result in faster delivery of a pre-determined BH dose. Because boiling initiation is required within each pulse, higher pulse average output power must be used in these accelerated treatment regimes, so that the boiling temperature is reached within the shorter pulses.

The feasibility and relative efficiency of this treatment acceleration strategy for transcutaneous BH *in vivo* was evaluated by producing volumetric lesions in renal cortex with pulse durations of 1, 2, 5 and 10 ms at the pre-defined dose of 10 pulses/focal spot in 3 exposures each. Table 1 lists the pulsing parameters for all treatment regimes. The pulse average acoustic power required for treatment initiation for each regime was determined experimentally from the appearance of a hyperechoic region at the focus; as seen, the power requirements increased almost 4-fold with when pulse duration was reduced from 10 ms to 1 ms. Note that this treatment acceleration strategy was only tested in renal cortex, not medulla and collecting system, because fractionation in these tissues was not complete in any of the exposures, and it was therefore impossible to define and quantify contiguous lesion volume.

**Table 1:** Parameters of the BH protocols used in the testing of treatment acceleration strategy in kidney cortex.

Pulse duration, ms	PRF, Hz	Peak acoustic power, kW
10	1	0.88–1.5
5	2	1–1.8
2	5	1.3–2.8
1	10	3.5–3.8

**Figure 5:**
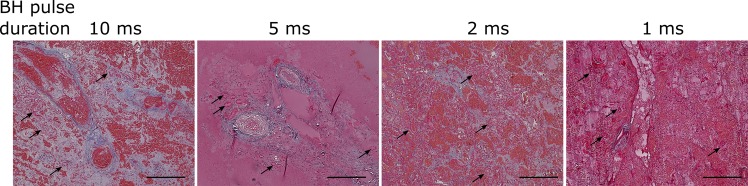
Operating at higher peak power outputs and shorter pulses accelerates BH treatments. Histological slides of the volumetric lesions stained with Masson’s trichrome stain in kidney cortex for low dose BH exposures consisting of 10 pulses of progressively shorter duration (as indicated) delivered at increasing pulse repetition frequency (duty cycle was constant and equal to 1%). As the pulse duration was decreased, peak acoustic output power was increased to achieve boiling within each pulse (Table 1 and Fig. 9). The degree of liquefaction was the same across all exposures, while accelerating the treatment 10-fold. At this lower dose, some connective tissue fragments and structures with adjacent cells (black arrows) were observed within the lesions. The scale bar is 250 microns.

All treatments were feasible at the depths of 20–40 mm from the skin (10–15 mm in kidney) and produced homogenized voids with the shape conforming closely to the planned ablation volume, to within the resolution of post-treatment ultrasound imaging (0.7 mm axial and lateral resolution at the depth of interest, according to the probe specification). The volume of the lesions was determined from post-treatment ultrasound imaging and was 0.136 ± 0.06 cc, whereas the overall treatment time decreased proportionally to PRF. The use of the shortest pulse duration of 1 ms resulted in the lysis rate of 19.6 ± 8 cc/hour, and the longest pulse duration of 10 ms – 1.9 ± 0.8 cc/hour. Qualitatively, the same degree of cellular fractionation was achieved with all four pulse regimes producing histologically similar cortical lesions (Fig. 5).

### Feasibility and safety of liver treatments

**Figure 6:**
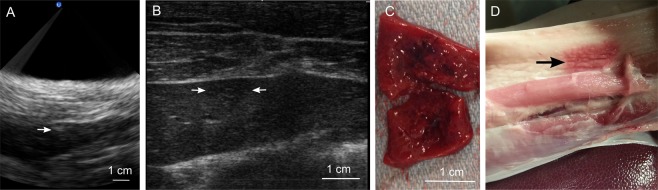
Investigation of feasibility and safety of volumetric BH treatment of porcine liver. (**A**) B-mode ultrasound image guidance during treatment of the liver. The white arrow indicates the location of the hyperechoic region at the focus, which was much less discernible than that in kidney treatments. This is likely due to the aberrating effects of fat-containing body wall overlying the liver. (**B**) Post-treatment ultrasound evaluation of the treated area. The hypoechoic region of ~1 cm size corresponding to the liquefied area is marked by the two white arrows. As seen, the liquefied region was almost indiscernible in liver and much less defined than that in kidney. This could be due to the aberrating effects of the body wall degrading image quality and/or due to the incomplete loss of structure and retention of the lobular compartments in the ablated area (see below). (**C**) A representative gross view of the bisected liquefied lesion in the liver. (**D**) A representative gross view of the body wall overlying the treated liver. The arrow marks what appears to be a mix of mechanical and thermal damage to the fatty layer of the body wall. This collateral damage was observed in three out of the four treated livers.

Figure 6 summarizes the observations pertaining to BH liver ablations. Subcostal BH lesions (N = 10) were successfully produced in four livers in which treatment was attempted using the same pulsing protocol as above (10-ms pulses, 20 pulses per focal spot, pulse repetition frequency 1 Hz) with focal spots spaced by 1.5 mm. The threshold for initiating BH transcutaneous subcostal treatment in liver was 1200–3600 W, i.e. 3-9-fold higher than in *ex vivo* liver (400 W) and was approaching the output limit of the driving electronics. The threshold was also larger than in the transcutaneous subcostal kidney exposures despite very similar overall depth in tissue and body wall thickness. However, the portion of body wall in the beam path contained a much thicker on average (1.5 cm vs 0.5 cm) layer of subcutaneous fat for liver ablation vs. kidney ablation. As fat is known to cause significant aberrating effects, we hypothesized that this contributed greatly to these differences. In addition, the hyperechoic region appearing on ultrasound imaging during treatment, as well as the hypoechoic region post treatment was much harder to discern compared to kidney treatments (Fig. 6A,B). This was also attributed to the aberrative effects of fat within the body wall that is known to degrade ultrasound image quality^31^. The respiration-induced motion of the target was much more pronounced compared to the case of kidney treatments, and led to a noticeable spread of the lesion relatively to the planned shape. The hepatocytes in the central region of the lesion were completely homogenized, while at the lesion periphery the treatment effect was less demarcated. In seven out of ten successful treatments, bruising and thermal damage confined to the fatty layer of the body wall immediately overlying the liver were observed (Fig. 6D). No other collateral damage was noted.

### Histological assessment of liver BH ablation

**Figure 7:**
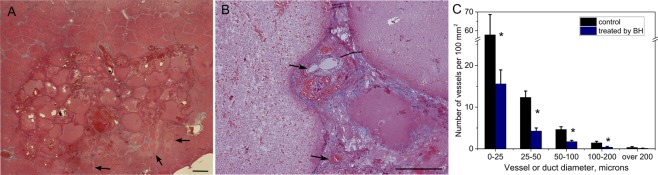
Histological analysis of connective tissue structures within volumetric BH lesions in porcine liver. (**A**) Masson’s trichrome (MT) stained histological section of a liquefied region in the liver. The lobular structure of the liver and the blood vessels and bile ducts are retained within the lesion, although the tissue within each lobule is liquefied. Narrow regions of thermal damage adjacent to the liquefied area (arrows) were observed in some of the lesions as more eosinophilic areas (stained darker pink) that form due to desiccation of cells. The scale bar is 1 mm. (**B**) Higher magnification histological view of blood vessels within the lesion (arrows). (**C**) Size distribution of the connective tissue structures (blood vessels and ducts) within intact liver tissue and BH-liquefied lesions. Error bars represent standard deviation; *significant difference between treatment and control group per Student’s t-test at p < 0.05.

*In vivo* BH ablation of liver produced completely homogenized hepatocytes while sparing the fibrous lobule boundary (Fig. 7A). Additionally, the liver capsule, blood vessels and biliary ducts were retained within the liquefied lesion (Fig. 7B). Larger caliber vessels and ducts appeared to be more resistant to BH damage. Specifically, there was a statistically significant 3–4 fold difference in the number of structures of <200 micron size between BH-treated lesion and intact liver tissue, but no difference in the number of vessels and ducts over 200 microns in size (Fig. 7C). These findings are consistent with prior observations of histotripsy damage by our group and others^20,22^. Narrow regions of thermal damage adjacent to the mechanically fractionated tissue, distal with regards to the HIFU transducer, were noted in 2 out of 4 livers treated (see Fig. 7A). This is most probably due to the application of high-amplitude pulses that did not induce boiling within the 10 ms pulse, but were absorbed at the focus causing thermal damage. This effect was observed before in the cases when boiling was not initiated^30^.

### *Ex vivo* investigation of aberrative properties of fatty layers

**Figure 8:**
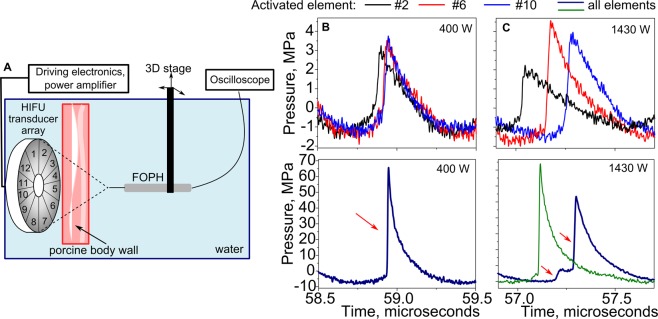
*Ex vivo* investigation of the aberrative properties of fatty layers. (**A**) Schematic of the experimental setup. A 3–4 cm thick segment of the body wall overlying the liver, consisting of alternating layers of fat and muscle of varying thickness, was freshly collected from the experimental animals. Fiber Optic Probe Hydrophone (FOPH) was used to record ultrasound waveforms at the HIFU transducer focus in free field in water and behind the segment of the body wall. (**B**) Representative focal waveforms in shock forming regime (acoustic power 400 W) produced by individual elements and all elements of the array measured in water in free field. Each element of the array produced a small amplitude shock wave at the focus, and the shock fronts were temporally aligned. When all elements were activated simultaneously, a shock front of sufficient amplitude for BH ablation was formed at the focus (red arrow). (**C**) Representative focal waveforms from individual array elements and the entire array recorded in water behind a 3 cm thick segment of the body wall at higher acoustic power (1430 W) that was expected to compensate for tissue attenuation (1.7 dB/cm). Although the amplitude of the signals from individual array elements was similar to that measured in free field as expected, the shock fronts were not phased-in. This is likely due to the difference in propagation time through different regions of the body wall that corresponded to the positions of the elements. When the entire array was activated, the focal waveform (bottom, blue line) contained multiple shock fronts (red arrows) of smaller amplitude, that would preclude the initiation of boiling within the BH pulse. When phase delays were introduced for individual array elements based on the FOPH measurements, the shock front was restored to its expected amplitude (green line). It is thus concluded that the main limiting factor in performing extracorporeal BH is aberration induced primarily by fat layers. These measurements were performed four times with similar results.

To investigate the cause of the high power levels required to perform BH ablation in liver, body wall segments immediately overlying the liver of N = 4 animals were collected and used in *ex vivo* investigation of their effect on the HIFU field. Figure 8 summarizes the measurement arrangement and the major findings. First, focal waveforms produced by each of the twelve HIFU array elements separately, and by the entire array were measured in water by fiber optic probe hydrophone (FOPH) at the shock-forming acoustic output power (400 W). As seen in Fig. 8B, each HIFU-array element produced a small-amplitude shock front of about 2–3 MPa at the focus, and the shock fronts arrived at the focus nearly simultaneously, i.e. were phased in. When all array elements were simultaneously activated, a waveform containing shock amplitude sufficient for BH initiation was produced at the focus. Then, a body wall segment was inserted between the transducer body wall did not substantially change the amplitudes of the focal waveforms from separate elements, but introduced relative delays to the arrival times of the shock fronts within 200–300 ns interval. This de-phasing lead to splitting of the shock front in the array focal waveform into 2–3 shock fronts of reduced amplitude that would not be sufficient for initiation of boiling within a 10-ms pulse. Importantly, this aberrative effect could be corrected for by introducing phase delays for each array element according to the hydrophone measurements; in that case, the shock front amplitude was restored to that measured in free field conditions (Fig. 8C).

## Discussion

In this work, transabdominal and partial transcostal volumetric BH ablation of the kidney and liver was realized in a large animal model for the first time. These data indicate that BH mechanical ablation is feasible in both organs, provide insight on how to further refine the technology to ensure safety and efficacy for clinical implementation, and suggest a metric for BH dose standardization.

In kidney, all treatment protocols produced the expected tissue effects and appeared safe, with no observable side effects on intervening tissue. Delivering higher amplitude pulses of shorter duration at higher PRF and constant duty cycle allowed for more rapid, yet equally efficacious tissue homogenization for a given pulse number/focus. The highest ablation rate achieved in kidney was 19 cc/hour, which would make this technique amenable to treating most small renal masses (clinically defined as ≤4 cm) in 2 hours or less, well within a clinically acceptable time frame. Since the degree of tissue homogenization, qualitatively, was found to be similar for all pulsing protocols or total “HIFU on” time, but differed depending on the number of BH pulses delivered per focus, the latter parameter was considered to be the best candidate for BH dose metric. In the case of volumetric ablation, this metric also quantitatively depends on spacing between focal sites, which in this work was determined empirically based on our prior experience with single lesions and the requirement for lesion merging.

In liver, volumetric BH lesions were successfully generated but required substantially higher output power to initiate the treatment compared to kidney despite treating at the same depth. We hypothesize that the increased power requirement was related to the anatomical features of pigs in that they have a thicker layer of inhomogeneous subcutaneous fat in the epigastrium (upper anterior abdominal wall) compared to the flank. Consequently, liver sonications penetrated more attenuating fat vs. exposures targeting the kidneys. In subsequent *ex vivo* experiments, we observed that aberration of the HIFU beam by this inhomogeneous fat was the main mechanism that reduced the *in situ* shock wave amplitude and thus prevented BH treatment initiation. In humans unlike pigs, subcutaneous fat tends to be greater in the flanks compared to the anterior abdomen. Additionally, with obesity, humans develop significant para and peri-nephric fat surrounding the kidney. Consequently, the impact of fat attenuation may be a greater challenge in renal BH than in liver BH clinically as has been reported during clinical thermal HIFU treatments of renal carcinoma^32^. However, introducing phasing delays was able to overcome the aberrative effects of fat *ex vivo* suggesting that strategies for aberration correction will overcome the challenges of fat aberrations and allow for improved treatment efficiency.

The feasibility of transcostal BH ablation was also evaluated in kidney by intentionally obstructing up to about 40% of the HIFU beam by ribs in 4/28 kidney exposures. Output power required to initiate BH treatment in these cases was 1.7–2.2 times higher than transabdominal exposures in which the beam path was unobstructed. As previously shown, the presence of ribs not only blocks the corresponding part of the beam power but also introduces aberration effects which result in distortion of the spatial pattern of the beam and further decrease of the focal pressures^33–35^. The contribution of such aberration effects is particularly strong for large aperture transducers when the beam passes through a periodic structure of several ribs, producing focus splitting with additional two-fold decrease of the focal intensity^36^. However, in the present experiments with relatively small transducer, only one or two ribs of the size comparable to the beam width interfered with the beam without creating a periodic structure. Therefore, strong focus splitting effects were not expected. Importantly, no side effects were grossly observed on or around the ribs located in the beam path during transcostal exposures, unlike commonly reported damage during thermal HIFU ablation. This is likely due to the short pulses and low duty cycle used for BH exposures; the potential for mechanical damage similar to that reported for shock wave therapies is mitigated by the ribs location in the near field, away from the main focal lobe^37^. Similarly, no adverse effects on the ribs were observed in prior studies on cavitation-based histotripsy^19,22^.

Standard B-mode ultrasound imaging, without synchronization with BH treatment, was sufficient for providing treatment targeting and real-time monitoring in kidney. The initiation of treatment could be seen easily as the appearance of hyperechoic bubbles at the focus, while the treatment outcome could be clearly visualized by the development of a hypoechoic region during all kidney treatments. As for imaging BH treatments in liver, because of the more pronounced imaging beam aberrations in the overlying fatty layer and more pronounced respiratory motion, treatment guidance and observation of the outcomes were degraded. One method to improve the sensitivity of detecting vapor bubbles at the focus in the presence of aberrations could be the use of plane wave Doppler ultrasound imaging combined with B-mode ultrasound and synchronized with BH pulse delivery, as previously reported for cavitation bubbles^38^. Improved means of visualizing the liquefied cavity post treatment could be based on the change of tissue stiffness rather than loss of echogenicity, which may not be very pronounced in some cases. Acoustic radiation force imaging (ARFI) or shear wave elastography have been clinically used as ultrasound-based methods of visualizing tissue stiffness distribution^39^ and some aspects of these methods have been shown to be useful in characterizing the effects of cavitation-based histotripsy treatments^40,41^.

*In vivo* renal tissues showed differential tissue specific sensitivities to equal BH doses with collecting system being least susceptible, followed by medulla and then cortex, confirming our previous work *ex vivo*^42^. Similarly, in liver larger caliber vessels and biliary structures in portal areas demonstrated lower susceptibility to BH than hepatocytes and small caliber central veins contained within the lobules also consistent with our previous *ex vivo* experiments^23^. Tissue specific sensitivities are likely due to differences in collagen content between tissue types and associated differences in mechanical strength and elastic modulus^43^. Clinically, these differences in tissue specific sensitivities could be advantageous in helping to reduce complications (such as urinary fistula and bile leaks). Additionally, within the kidney, human renal carcinoma has decreased collagen expression^44^ compared to normal renal parenchyma suggesting increased sensitivity to BH for renal carcinoma compared to normal kidney. If confirmed, increased sensitivity of renal carcinoma over normal renal parenchyma would help ensure effective destruction of tumor while facilitating nephron sparing in order to preserve patient renal function.

While these initial *in vivo* transcutaneous results are encouraging, we did observe some side effects of transcutaneous BH treatment. Following renal BH, we observed small areas of petechial hemorrhage in the region prefocal to the ablation zone within 1–2 mm. We hypothezise that this could be due to a combination of pre-focal caviation in front of the BH bubble cloud^30^ and/or the periodic shear stress from the radiation force produced by the incoming HIFU pulses causing mild, sub-lethal bruising of the kidney. The extent of this side effect is comparable to that seen in partial nephrectomy adjacent to the resection bed due to handling and manipulation of tissue. The other observed side effect was the presence of small clots in the collecting system of 8/11 treated kidneys. This is likely caused from extravazated erythrocytes traveling through the distal tubules and collecting ducts into the collecting system and aggregating. Clinically, this would likely be manifested as limited gross hematuria, which we anticipate would resolve in <24 hours based on prior rat studies performed by our group^29^. The use of much higher output power for sonicating liver led to prefocal thermal damage of the intervening fat layers in most subjects. Narrow areas of thermally ablated liver tissue were also observed adjacent to the liquefied lesion, indicating that some of the delivered BH pulses did not contain sufficient shock amplitudes to reach the boiling temperature within the 10 ms pulse and instead caused thermal denaturation within the narrow focal area. As outlined above, we hypothesize that phase correction strategies that help overcome the aberrative effects of tissue inhomogeneities will help alleviate these unwanted thermal effects. In addition, treatment precision in liver was more affected by the respiratory motion than in kidneys, resulting in irregular tissue fractionation at the lesion borders. In the future, respiratory gating of BH pulses when needed will likely mitigate the effects of respiratory motion.

In conclusion, these results demonstrate the feasibility and system requirements for transcutaneous and transcostal BH mechanical ablation of abdominal targets such as kidney and liver. BH dose-dependent tissue sensitivities with resulting selective tissue sparing may improve the efficacy of renal tumor ablation, while facilitating sparing of normal tissue to maintain organ function and reducing the risk of structural complications, thereby improving the safety of BH ablation. Finally, this study identified several challenges in BH ablation to be addressed to facilitate clinical translation, namely HIFU beam aberrations and respiratory motion during treatment. Work is currently ongoing to further refine the technology and address these challenges and to assess the tolerability of BH ablation in chronic large animal studies.

## Methods

### Experimental procedures

All procedures in the animal experiments followed the protocols approved by the Institutional Animal Care and Use Committee at the University of Washington, and all experiments were performed in accordance with relevant guidelines and regulations. The animals were housed in a facility at the University of Washington that is fully accredited by the Association for Assessment and Accreditation of Laboratory Animal Care International. The animals were cared for by a full-time veterinary staff. Before each experiment, the swine (37–40 kg, N = 6) was anesthetized with Telazol premedication, then masked with isoflurane and intubated. Pigs were placed on the surgical table in either lateral (for kidney treatment) or supine (for liver treatment) position. The skin overlying the kidneys and liver was cleaned, shaved and depilated. B-mode imaging (Ultrasonix, RP Acoustics, Toronto, Canada) of the targeted area was performed prior to and following BH exposure with a high resolution linear imaging array (Ultrasonix L14–5, operated at 6.6 MHz frequency). In particular, during the post-treatment imaging each volumetric lesion was continuously hand-scanned in two orthogonal directions corresponding to the directions of transverse HIFU focus translation during treatment. Cine-loops of each scan were recorded, and then still images of the lesion in the two orthogonal imaging planes were taken in the center of the lesion. Multiple BH exposures were administered to each kidney and liver under real-time ultrasound imaging guidance using a different imaging probe - lower frequency, smaller aperture phased array (Ultrasonix PA7-4, aperture size 10 × 15 mm) operating in B-mode, under tissue harmonic imaging setting (using second harmonic echoes of the 4 MHz center frequency for image formation). The reason for using two different probes for pre- and post-treatment imaging was primarily due to the limitations imposed on the array used during treatment: its aperture was determined by the size of the HIFU transducer central opening and had to be very small, while the available imaging depth had to be fairly large to account for the distance between the array surface and the HIFU focus. The PA7-4 phased array used during treatment provided a reasonable tradeoff between image quality, that was degraded due to the limitations listed above, and our ability to target areas of kidney and liver and detect hyperechoic bubble activity. After the post-treatment ultrasound imaging, necropsy was performed and the treated portions of the liver and the kidneys were collected for gross and histologic assessment.

### HIFU transducer and pulsing protocol

A 1.5 MHz HIFU transducer (12-element sector array of 7.5 cm aperture, F# = 1.07) with a central opening (2 cm diameter) that accommodated an ultrasound imaging probe (PA7-4) for real-time guidance was attached to a 3D positioning system and submerged in a degassed water bath coupled to the abdomen (Fig. 1). The HIFU focus position, pre-recorded with the ultrasound imaging system, was aligned with the targeted region at the depth of 1.5–4.5 cm from the skin surface. The length of the focal region of the transducer measured in water at -6 dB level in linear regime was 14 mm and the width was 1.6 mm^45^. Custom-built high-power electronics were used to drive the transducer within the acoustic peak power range between 1–3.8 kW^46^. The pulse-average power output threshold for initiating BH at each location was measured by sonicating a single focal point (the first point in the treatment grid) with isolated BH pulses at gradually increasing amplitude until a hyperechoic region was observed at the focus, indicating boiling. The volumetric treatments that followed were performed slightly above the threshold (10–15% increase in driving voltage), with all points in the treatment grid.receiving the same power. Prior to the *in vivo* experiments, similar measurements of threshold output power were performed in freshly harvested *ex vivo* porcine liver and kidney for comparison to the transcutaneous *in vivo* setting.

### Hydrophone measurements

**Figure 9:**
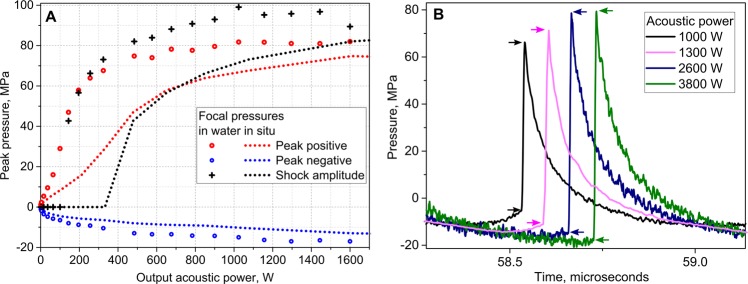
Acoustic pressure levels at the focus used in the experiment. (**A**) Peak pressures and shock amplitude provided by HIFU array transducer at the focus in water (symbols) and derated into tissue (dashed lines) versus peak output acoustic power. Tissue attenuation was considered as 1.7 dB/cm, depth in tissue 3 cm. (**B**) Representative derated acoustic pressure waveforms used in experiments aimed at BH treatment acceleration; increasing peak acoustic output power was used for exposures with pulse durations of 10, 5, 2, and 1 ms. Lower and upper boundaries of the shock front are indicated by arrows; with increasing output power, the shock amplitude changed relatively to peak pressures from being nearly equal to peak positive pressure to being close to peak to peak pressure. Duty cycle was kept constant and equal to 1% in all exposures.

Total acoustic power produced by the HIFU transducer within acceptable input power limits was measured by acoustic radiation force balance^47^. The focal pressure waveforms corresponding to those acoustic power levels were measured in water by the fiber optic probe hydrophone (FOPH2000, RP Acoustics, Leutenbach, Germany) and the results are presented in Fig. 9A. The attenuation of the body wall segments was estimated as the ratio of focal pressures in the linear propagation regime with and without body wall in place, and was consistent with prior findings – 1.7 ± 0.2 dB at 1.5 MHz^46^. To estimate the focal pressures *in situ*, the peak pressures measured in water were derated to 3 cm depth in tissue with attenuation of 1.7 dB/cm using a modified derating approach for nonlinearly distorted waveforms as previously described^24,48^. Figure 9B shows the representative derated focal waveforms used in experiments aimed at BH treatment acceleration in kidney cortex; increasing peak acoustic output power corresponded to the pulse durations of 10, 5, 2, and 1 ms. The lower and upper boundaries of the shock front are indicated with arrows in Fig. 9B and were determined at the level of 2.5% from the maximum of the time derivative of the pressure waveform, according to the definition developed previously^49^. The increasing *in situ* shock amplitudes corresponds to progressively shorter time to reach boiling temperature, estimated from weak shock theory as 5, 4, 3 and 2 ms, respectively^50,51^.

### Histology

The treated regions of liver and kidney were carefully resected *en bloc* immediately post animal euthanasia and fixed in 10% neutral buffered formalin, followed by processing and paraffin embedding for histological analysis. Two serial histological sections of 5 μm thickness were taken every 500 μm throughout the sample in the plane parallel to the HIFU propagation direction. The consecutive sections were stained with hematoxylin and eosin (H&E) and Masson’s trichrome stain. To quantify the distribution of sizes of blood vessels and bile ducts in the histological sections of intact liver tissue vs BH lesions (Fig. 7C) the following procedure was used. A Masson’s trichrome-stained section corresponding to the core of each lesion was selected. Between 20–135 10x images per slide (control or BH-treated) were taken; the images were evaluated for vessels and ducts, and the minimum ferret diameter of each vessel was measured by hand in ImageJ. The number of vessels was normalized by 100 mm^2^ and grouped by the size range. Student’s t-test was used to compare treated and control groups within each size range. P-values < 0.05 were considered statistically significant.

### Transmission electron microscopy (TEM)

A set of 5 mm wide BH lesions produced in kidney cortex of one animal and resected *en bloc* were placed in ½ strength Karnovsky’s fixative. The tissue was sliced into 1 mm sections parallel to the direction of treatment. Small regions (1 × 2 mm) from the grossly identified lesions and their border were sampled and processed for transmission electron microscopy (TEM) as previously described^23^. Briefly, fixed samples were stained with 1% OsO4 in phosphate buffered saline (PBS), then dehydrated in ascending acetone concentrations and embedded in EPON-Araldite mixture resin. After polymerization, 80 nm thick sections were cut using an LKB ultramicrotome. Sections were placed on carbon coated Cu grids and stained with lead citrate according to published procedure^52^. Grids were visualized using a JEOL JEM-1011 transmission electron microscope (Jeol USA Inc, Peapody, MA, USA). Representative images were taken at 80 kV using a GATAN SC1000W camera with 8.5 Mpixels resolution (Gatan Inc, Pleasonton, CA, USA) and Micrograph software.

## Supplementary information

Supplementary figures.

## Data Availability

The datasets generated during and/or analyzed during the current study are available from the corresponding author on reasonable request.
