# Supplementary material for: Pilot in vivo studies on transcutaneous boiling histotripsy in porcine liver and kidney
Source: Sci Rep. 2019 Dec 27;9:20176. doi: 10.1038/s41598-019-56658-7 (PMC6934604; doi:10.1038/s41598-019-56658-7)
Supplement: Supplementary file 1 — Supplementary figures. [file 41598_2019_56658_MOESM1_ESM.docx]

**Supplementary Information – Supplementary Figures**

**Pilot *in vivo* studies on transcutaneous boiling histotripsy in porcine liver and kidney**

**Tatiana Khokhlova^1,2*^, George Schade^3*^, Yak-Nam Wang^2^, Sergey Buravkov^4^, Valeriy Chernikov^5^, Julianna Simon^2^, Frank Starr^2^, Adam Maxwell^3^, Michael Bailey^2^, Wayne Kreider^2^, Vera Khokhlova^2,6^**

^1^ Division of Gastroenterology, Department of Medicine, University of Washington, Seattle. WA, USA

^2^ Center for Industrial and Medical Ultrasound, Applied Physics Laboratory, University of Washington, Seattle, WA, USA

^3^Department of Urology, University of Washington, Seattle. WA, USA

^4^ Faculty of Fundamental Medicine, M.V. Lomonosov Moscow State University, Moscow, Russia

^5^Research Institute of Human Morphology, Moscow, Russia

^6^ Physics Faculty, M.V. Lomonosov Moscow State University, Moscow, Russia

*First two authors (Tatiana Khokhlova and George Schade) contributed equally to this work

*Corresponding author: Dr. Tatiana Khokhlova (email: tdk7@uw.edu)


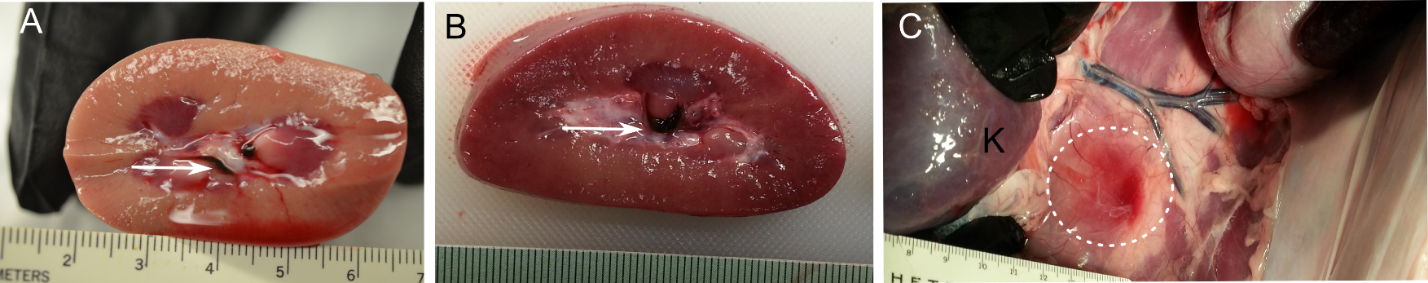


**Supplementary Figure S1 A, B** Small blood clots (arrows) were observed in the collecting system of 8/11 treated kidneys, regardless of which part of the kidney was targeted (e.g. cortex, medulla or collecting system). **C.** Subtle bruising of the side wall (dashed circle) adjacent to the kidney (K) following superficial BH treatment of the cortex.


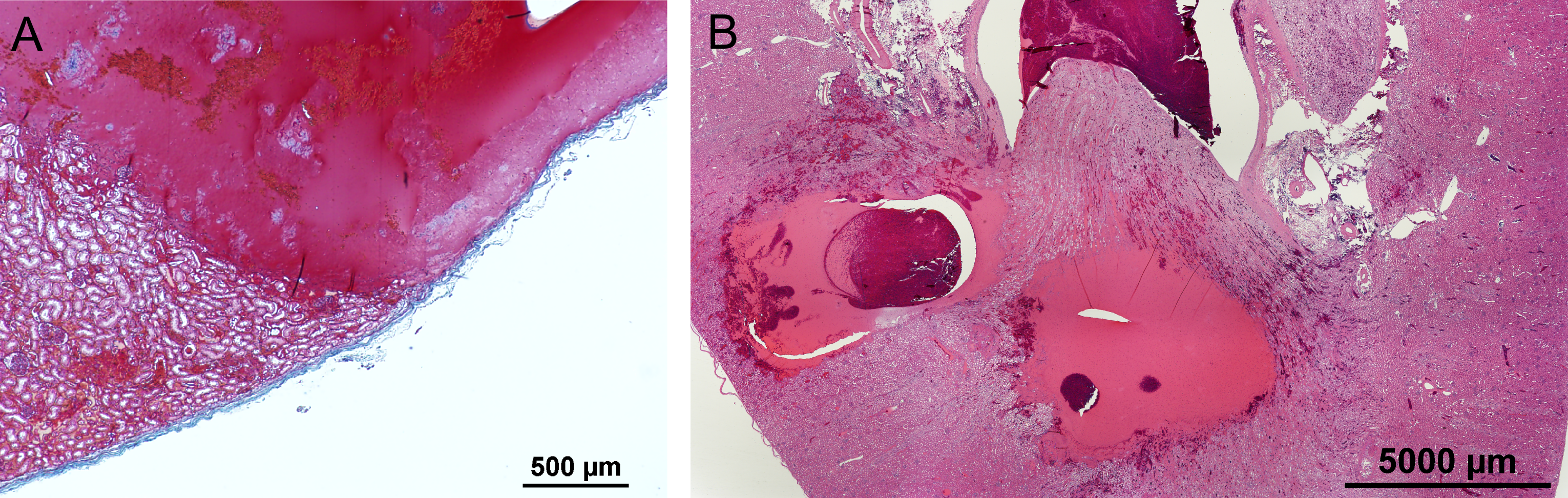


**Supplementary Figure S2. A.** Masson’s trichrome stained histological slide of a BH lesion produced immediately adjacent to kidney capsule which stayed intact. **B.** H&E-stained histological slide of two volumetric lesions produced in kidney cortex, separated by a 1 mm gap, illustrating the precision of BH treatment.


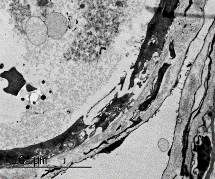

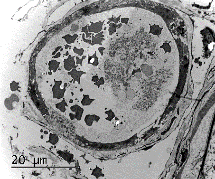

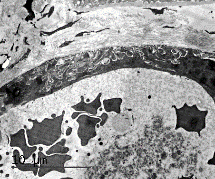

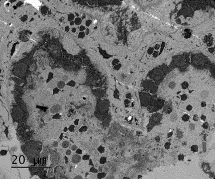

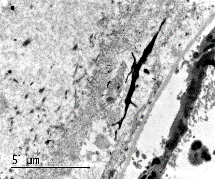

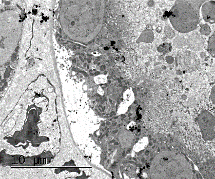


**A**

**B**

**C**

**D**

**E**

**F**

**Supplementary Figure S3a. Representative TEM images of renal cortex partially damaged by BH**.

**A, B** - Ruptured basal membranes of proximal renal tubules (arrows). **C** - Partial delamination of basal plasma membrane (arrows). **D** - Separation of cells from basal membrane (arrows). **E** – basal plasma membrane, echinocytes, and lipid droplet (arrows) inside the intact part of a tubule. **F** –collagen fibers and fibroblasts (arrows) appear intact outside tubules while cells of basal plasma membrane is delaminated from basal membrane (arrows).


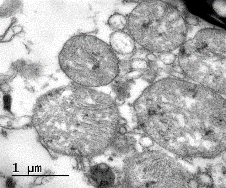

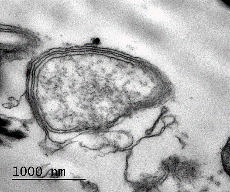

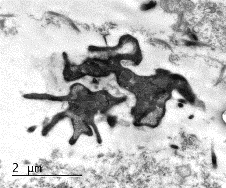

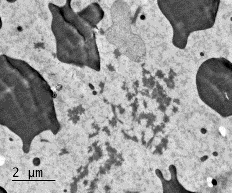

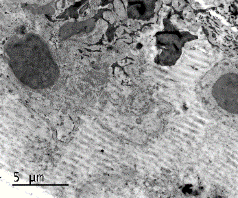

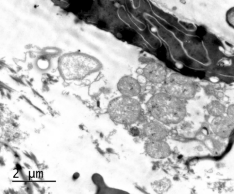


**A**

**B**

**C**

**D**

**E**

**F**

**Supplementary Figure S3b. Representative high magnification TEM images of incomplete tissue defragmentation close to the lesion border in renal cortex. A – C -** Fragment of basal plasma membrane (red arrow), swollen mitochondria without crists outside cells (blue arrows), and multilamilar bodies with completely fragmented lesion content (green arrows). **B** and **C** are enlarged regions of **A**. **D – F** – cell debris (red arrow), cell nucleus (blue arrow), echinocytes and collagen debris (green arrows) inside the lesion.
